# Successful treatment of annular lichenoid dermatitis of youth with topical ruxolitinib

**DOI:** 10.1016/j.jdcr.2025.07.012

**Published:** 2025-08-06

**Authors:** Aleia Boccardi, Paras Patel, Rebeca Teplitz, Mark Jacobson, Suzanne Friedler

**Affiliations:** a Department of Dermatology, St. John’s Episcopal Hospital, Far Rockaway, New York; b Service, Veterans Affairs New York Harbor Healthcare System, Brooklyn Campus, Brooklyn, New York; c Rowan University School of Osteopathic Medicine, Stratford, New Jersey

**Keywords:** annular lichenoid dermatitis of the youth, ALDY, Janus kinase inhibitors, JAK inhibitors, ruxolitinib

## Introduction

Annular lichenoid dermatitis of youth (ALDY) is a rare, asymptomatic dermatosis initially reported in adolescents of Mediterranean heritage.^1^ Since its discovery in 2003, while there have been less than 100 cases reported, it has now been seen across all age groups and more geographic locations including Europe, Japan, India, United States, and Korea.^1^, ^2^, ^3^, ^4^ Clinically, ALDY features are nonspecific, presenting as red-violaceous to brown annular plaques with central hypopigmentation. Consideration and exclusion of dermatoses such as autoimmune connective tissue diseases, annular erythemas, and mycosis fungoides (MF), along with careful clinicopathologic correlation is vital in accepting the diagnosis.^1^, ^2^, ^3^, ^4^, ^5^ Currently, no standardized treatment exists, and no single therapy has demonstrated consistent efficacy.^1^ We present a unique case of ALDY successfully treated with a topical Janus kinase (JAK) inhibitor.

## Case report

A 28-year-old Caucasian female presented to our dermatology office with 2 discolored skin lesions on her abdomen and back for approximately 4 months. Her past medical history included idiopathic urticaria, angioedema, and pyogenic sacroiliitis and osteomyelitis leading to pyomyositis of the iliopsoas muscle–acquired and treated with prolonged antibiotics during childhood. She was not on any medications at the time of presentation and reported a family history of Sjögren’s syndrome and lupus in her mother and maternal aunt, respectively. The rash was nonpruritic, nontender, and described as varying in color from red/violet to light brown. She experienced no extracutaneous symptoms and denied similar skin eruptions in the past, recent travel, or contacts exhibiting a similar rash. Laboratory results for antinuclear antibody, RF, anticyclic citrullinated peptide, anticentromere, anti-Ro, anti-La, anti-smith, anti–double-stranded DNA, antinuclear ribonucleoprotein, anti-Scl-70, complete blood count, erythrocyte sedimentation rate, hepatitis panel, Quantiferon gold, and rapid plasma reagin, completed independently by a rheumatologist for lupus workup, were all within normal limits.

**Fig 1:**
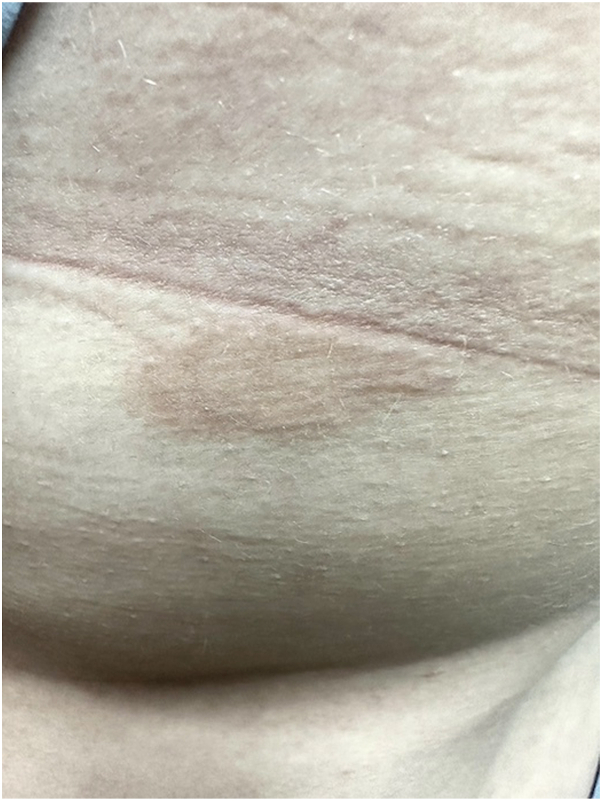
*Ovoid*, erythematous annular plaque with ill-defined central hyperpigmentation located on patient’s left lateral trunk, no secondary skin changes noted.

**Fig 2:**
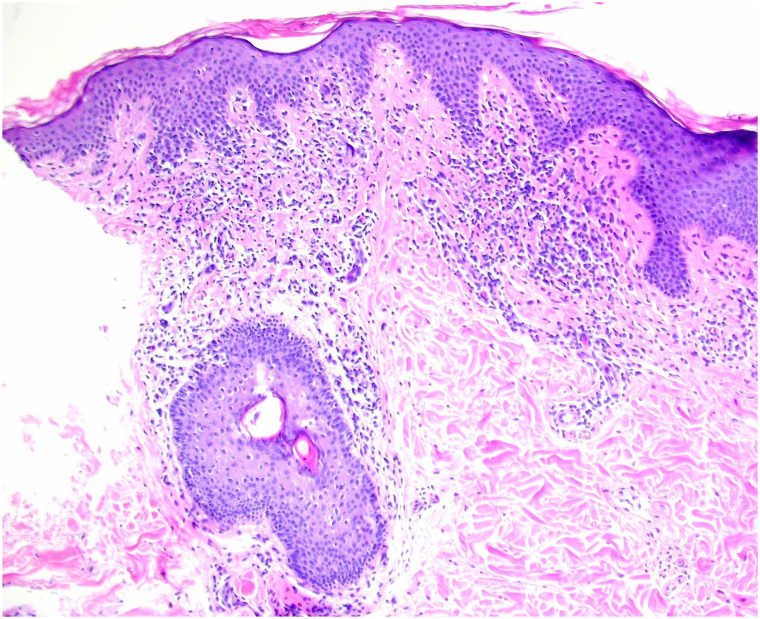
Punch biopsy showing vacuolar degeneration/apoptotic keratinocytes obscuring the tips of blunted rete ridges with a sparse lichenoid lymphohistiocytic infiltrate in the surrounding dermis.

Clinical observation revealed 2 discrete lesions. The first was erythematous, well-defined, and plaque-like located on the left periumbilical region. The second was a hyperpigmented, light brown, annular patch with central hypopigmentation on the posterior left lateral trunk (Fig 1). Neither presented with overlying secondary skin changes. A punch biopsy of the periumbilical site showed a superficial perivascular and lichenoid inflammatory infiltrate composed of lymphocytes, histiocytes, and occasional eosinophils that obscured the tips of blunted rete ridges where necrotic keratinocytes were confined (Fig 2). After clinicopathologic correlation and exclusion of other dermatoses, the diagnosis of ALDY was made. The patient was prescribed ruxolitinib 1.5% topical cream twice daily to affected areas. After 1 month of consistent use, she saw complete resolution and stopped the medication. No recurrence was noted at 2.5 months, after which she was lost to follow-up.

## Discussion

Understanding of ALDY has grown since its recognition 21 years ago, although its exact pathogenesis remains unclear.^1^, ^2^, ^3^, ^4^, ^5^ It is currently postulated to stem from a T-cell–mediated response, similar to other lichenoid skin reactions, but no environmental triggers (drugs, bites, allergies), infections, autoimmune conditions, or laboratory studies have been associated with the disease.^1^ Current debates revolve around a role for Borrelia and the potential of ALDY belonging to the morphea spectrum.^1^^,^^5^

ALDY is a diagnosis of exclusion. If suspicion for the condition arises, further investigation tailored to the patient should be completed to rule out more common and severe diseases such as autoimmune connective tissue diseases, hypopigmented MF, annular erythemas (Tables I and II), and morphea. Tests including antinuclear antibody with reflex, nontreponemal/treponemal tests, fungal culture/stains, hepatitis panel, quantiferon gold, etc. can help narrow the differential. Given our patient’s family history, clinical suspicion for annular erythema of Sjogren’s syndrome and cutaneous systemic lupus erythematosus took precedence. Aside from her negative autoimmune panel, since both conditions can present seronegatively, our patient did not exhibit symptoms suggestive of either or have lesions in typical annular erythema of Sjogren’s syndrome or cutaneous systemic lupus erythematosus distribution–face/extremities versus sun-exposed, respectively.^7^ Histopathology showed features consistent with ALDY, including a focal increase of vacuolar degenerated apoptotic keratinocytes at the tips of the rete ridges, along with less commonly seen sparse lichenoid infiltrate in the dermis (Fig 2).^2^, ^3^, ^4^, ^5^ Fungal stains (periodic acid-schiff, Grocott methenamine silver) and T-cell rearrangement study were not performed because the clinicopathologic correlations upon histologic analysis did not warrant further workup for dermatophytes or MF. Altogether, these findings led to accepting ALDY as our diagnosis. The patient was advised to complete follow-up appointments for continued monitoring.

**Table I:** Differential for common annular erythemas

Annular erythemas	Clinical appearance	Histological pattern	Associated symptoms	Known triggers	Diagnostic tests (most are clinical with aid of testing)
Erythema annulare centrifugum (EAC)	Superficial: urticarial erythematous plaque with trailing scale Deep: indurated erythematous plaque with cord-like border^6^	Superficial: spongiosis, parakeratosis, superficial perivascular lymphohistiocytic infiltrate^6^ Deep: mononuclear infiltrate at the mid-lower dermis^6^	+/− Pruritus	Fungal infections, Crohn’s disease, drugs, malignancy, idiopathic^6^	Microbiology, serology, and malignancy testing as needed
Erythema gyratum repens (EGR)	Concentric, erythematous, “wood-grained” plaque with scaling edges that spares the hands, feet, and face; rapid migration^6^	Focal parakeratosis, spongiosis, and superficial perivascular lymphocytic infiltrate with eosinophils^6^	PPK, hypereosinophilia, acquired ichthyosis	Malignancy (bronchial, esophageal, breast, stomach, GU, etc.), idiopathic, autoimmune disease (CREST, RA), drug, TB, PRP^6^	Quantiferon Gold, r/o malignancy, and serology (autoantibodies)
Erythema migrans	Erythematous annular/targetoid plaque with central clearing and centrifugal expansion; singular or multiple	Superficial and deep lymphoid infiltrates with eosinophils and plasma cells^6^	+/− Pruritus, pain, transient numbness	Tick bite transmitting *Borrelia burgdorferi*	Anti-Borrelia antibodies
Cutaneous lupus erythematosus	Erythematous polycyclic, annular plaques with crusted border and hypopigmented center	Vacuolar interface dermatitis with perivascular and periadnexal lymphoid infiltrate, hyperkeratosis, and mucin	Arthritis/arthralgias, systemic lupus symptoms	Photosensitive drugs (HCTZ, terbinafine, TNF-α inhibitors, PPI, CCB, etc.)	Autoantibodies, DIF
Erythema marginatum	Intermittent, erythematous annular and polycyclic, nonscaling plaques with migration	Perivascular infiltrate, predominantly of neutrophils^6^	Carditis, polyarthritis, chorea, subcutaneous nodules, swelling, nausea, vomiting, abdominal pain	Group A B-hemolytic streptococci (GAS) infection (pharyngitis), hereditary angioedema with C1-inhibitor deficiency^6^	Rapid GAS test, CBC, CRP, ESR, antistreptolysin O titer, C4, C1 esterase inhibitor, C1q^6^
Annular erythema of Sjögren’s syndrome	Erythematous annular/polycyclic plaque +/− scale with central clearing on sun-exposed areas	Superficial perivascular lymphocytic infiltration in a “coat-sleeve” pattern^7^	Xerophthalmia, Xerostomia, parotid gland enlargement, arthralgias	+/−Photosensitive^7^	Autoantibodies, salivary gland biopsy

*CBC*, Complete blood count; *CCB*, calcium channel blocker; *CRP*, C-reactive protein; *DIF*, direct immunofluorescence; *ESR*, erythrocyte sedimentation rate; *GU*, genitourinary; *HCTZ*, hydrochlorothiazide; *PPK*, palmoplantar keratoderma; *PPI*, proton pump inhibitor; *PRP*, platelet-rich plasma; *RA*, rheumatoid arthritis; *TB*, tuberculosis; *TNF-α*, tumor necrosis factor α.

**Table II:** Clinical features and laboratory markers comparing ALDY and annular erythemas

Distinguishing characteristics	ALDY	EAC	EGR	Erythema migrans	Erythema marginatum	cSLE	AESS
Clinical features							
Trailing scale	–	+	–	–	–	–	–
Wood grained/concentric	–	–	+	–	–	–	–
Annular plaque	+	+	+	+	+	+	+
Polycyclic	–	–	–	–	+	+/−	+/−
Scale	–	+	+	–	–	+/−	+/−
Centrifugal spread	–	+	+	–	–	–	–
Sun-exposed distribution	–	–	–	–	–	+	+/−
Systemic involvement	–	+/−	+/−	+/−	+	+/−	+/−
Laboratory markers							
Fungal culture	–	+/−	–	–	–	–	–
Group A strep	–	–	–	–	+/−	–	–
C4, C1 esterase, C1q	–	–	–	–-	+/−	+/−	–
Anti-dsDNA, antihistone, anti-smith	–	–	–	–	–	+	–
Anti-Ro/SSA	–	–	–	–	–	+/−	+
Anti-La/SSB	–	–	–	–	–	+/−	+/−
Anti-*Borrelia*	–	–	–	+/−	–	–	–
Quantiferon-gold	–	–	+/−	–	–	–	–

*AESS*, Annular erythema of Sjogren’s syndrome; *ALDY*, annular lichenoid dermatitis of youth; *cSLE*, cutaneous systemic lupus erythematosus; *dsDNA*, double-stranded DNA; *EAC*, erythema annulare centrifugum; *EGR*, erythema gyratum repens.

There are no clear algorithm or treatment guidelines for management of ALDY. Corticosteroids, calcineurin inhibitors, cyclosporine, antibiotics, and light therapies have all been tried with varying results.^1^^,^^4^ Due to the patient’s intensive past medical history and recent systemic lupus erythematosus workup, she was hesitant to start any topical steroid treatment, especially since she was asymptomatic. Therefore, we selected a trial of ruxolitinib for its convenient topical application, strong safety profile, and promising mechanism of action for lichenoid processes. Ruxolitinib is a JAK 1 and 2 inhibitor approved for the treatment of mild to moderate atopic dermatitis and vitiligo. It has been theorized ALDY is closely related to lichen planus (LP) and other lichenoid dermatologic conditions that are involved with the interferon (IFN) γ/CXCL10 pathway.^2^ Thought to increase the sensitivity of keratinocytes to inflammatory signaling, IFN-γ uses JAK/signal transducer and activator of transcription as a signal transducer to increase expression of CXCL10 and major histocompatibility complex 1.^8^ JAK inhibitors therefore can downregulate IFN-γ/CXCL10, resulting in reduction of inflammation and symptom resolution.^7^ Clinicians have begun to use this therapy to control LP. A literature review showcasing treatment efficacy of various JAK inhibitors against LP showed 73.3% of cases had partial or complete resolution.^8^ Specifically, ruxolitinib has been shown to improve appearance of the lesion, pruritus, and quality of life in 2 to 3 weeks, including patients with treatment refractory disease, without serious adverse events.^9^ Currently, a phase 2 trial is underway for treatment of oral LP with baricitinib.^10^

To our knowledge, this is the first reported successful treatment of ALDY with a topical JAK inhibitor, representing a novel approach to its management. Topical biologics continue growing in popularity and their vast potential is still being recognized. ALDY represents a rare disease for which randomized controlled trials are difficult to establish, but providers may consider ruxolitinib as a safe and effective treatment option.

## Conflicts of interest

None disclosed.
